# Serologic Testing of US Blood Donations to Identify Severe Acute Respiratory Syndrome Coronavirus 2 and Other Coronaviruses, December 2019 to July 2020

**DOI:** 10.1093/ofid/ofae351

**Published:** 2024-06-28

**Authors:** Kacie Grimm, Paula Saá, Narayanaiah Cheedarla, Michael S Gerty, Jamel A Groves, Roger Y Dodd, John Roback, Susan L Stramer

**Affiliations:** American Red Cross, Scientific Affairs, Rockville, Maryland, USA; American Red Cross, Scientific Affairs, Rockville, Maryland, USA; Department of Pathology and Laboratory Medicine, Emory University School of Medicine, Atlanta, Georgia, USA; American Red Cross, Histocompatibility Laboratory, Philadelphia, Pennsylvania, USA; American Red Cross, Scientific Affairs, Rockville, Maryland, USA; American Red Cross, Scientific Affairs, Rockville, Maryland, USA; Department of Pathology and Laboratory Medicine, Emory University School of Medicine, Atlanta, Georgia, USA; Infectious Disease Consultant, North Potomac, Maryland, USA

**Keywords:** blood donors, COVID-19, early seroprevalence, SARS-CoV-2, antibody detection

## Abstract

**Background:**

The first coronavirus disease 2019 (COVID-19) case in the United States was recognized on 19 January 2020, but the time of introduction of the virus into the United States is unknown. An existing sample cohort was examined for serologic evidence of early severe acute respiratory syndrome coronavirus 2 (SARS-CoV-2) infections.

**Methods:**

A repository of 46 120 samples from healthy routine blood donors, representing 46 states and the District of Columbia, was tested for total antibodies to SARS-CoV-2 nucleocapsid (anti-N) using a commercial test. All reactive samples were further tested using an experimental receptor-binding domain (RBD)–specific immunoglobulin G (IgG) enzyme-linked immunosorbent assay. Further testing was also conducted for anti-spike (anti-S) antibodies by commercial tests, experimental anti-S immunologic blocking, and for antibodies to the 4 human cold coronaviruses.

**Results:**

Anti-N reactivity was observed in 92 tested samples (0.2%), 91 of which had adequate volume for further testing; of these, 55 were confirmed positive by anti-RBD. None of these reactive findings were attributable to the other human coronaviruses tested. The confirmed-positive frequency increased over time paralleling patterns observed for COVID-19 cases reported in the United States (in contrast to stable patterns over time for the cold coronaviruses). Nine confirmed positive samples (0.07%) were identified among the 13 364 donations collected between 13 December 2019 and 22 January 2020. None of these early confirmed-positive samples were reactive by commercial anti-S tests suggesting very recent infection.

**Conclusions:**

The samples tested in this study were broadly representative of the United States, and all were from individuals who had successfully donated blood. The antibody-reactive results of this study suggest that SARS-CoV-2 was likely present in the United States before 19 January 2020.

## Teaser

SARS-CoV-2 infections in the US likely occurred before clinical COVID-19 disease cases. SARS-CoV-2 anti-nucleocapsid reactivity was low during the first 3 months (December 2019–March 2020), increased 10-fold in the following 3 months, and unrelated to the common cold coronaviruses.

The first clinical coronavirus disease 2019 (COVID-19) cases were identified in the United States on 19 January 2020, but the time of introduction for severe acute respiratory syndrome (SARS) coronavirus 2 (SARS-CoV-2) has not been definitively established [1, 2]. A subset (approximately 7400 samples collected in 9 states) of a large repository of routine whole-blood donor samples collected from 13 December 2019 to 17 January 2020 was used to estimate the early prevalence of SARS-CoV-2 using full-length spike (S) antibodies [3]. That study used a variety of alternate serologic tests to serve as confirmatory methods; however, no nucleocapsid (N) assays were used. The study found a frequency of 1.4% reactivity from 9 states in which the donors resided. Almost 96% of those with anti-S reactivity had further reactivity to the additional assays, suggesting that SARS-CoV-2 was likely present before 19 January 2020.

In the current study, the entire repository of >46 000 blood donation samples from 46 states and the District of Columbia was tested. In addition, a commercial anti-N assay was used, along with a series of commercial and research-based antibody assays, covering both SARS-CoV-2 N and S regions, for those that were initially reactive with the anti-N assay. The study covered a wider geographic area and longer time (to July 2020) than the prior study to evaluate the earliest antibody responses conclusively attributable to SARS-CoV-2 and increases in serologic reactivity over the first 6 months of the COVID-19 pandemic. We also assessed the reactivity of the SARS-CoV-2 antibody-positive samples to S and N antibodies of the common cold coronaviruses, SARS coronavirus (SARS-CoV), and Middle East respiratory syndrome coronavirus (MERS-CoV), to determine whether reactivity and trends could be attributed to those agents.

## METHODS

### Sample Repository

This repository included serum and plasma samples collected from consenting American Red Cross (ARC) donors from 13 December 2019 to early July 2020 (n = 46 120); all donors were healthy and reported that they were well and asymptomatic. The repository included samples from all 4 US census regions, with higher frequency of samples from the Midwest and Northeast regions [3]. Samples were tested for SARS-CoV-2 antibodies using commercial and research-based serologic assays. The creation of the repository and its use for SARS-CoV-2 studies were approved by the ARC Institutional Review Board. Donors provide written consent for blood donation and suitability testing and are provided an information sheet describing the possible uses of their data and/or residual samples.

### SARS-CoV-2 N Antibody Screening

The sample repository was initially screened on the cobas e601 analyzer using the Elecsys Anti-SARS-CoV-2 electrochemiluminescence immunoassay (ECLIA) (Roche Diagnostics; reference 09203095190), a qualitative total immunoglobin assay. This assay uses recombinant proteins representing the N antigen to detect SARS-CoV-2 antibodies, with an overall specificity of 99.81% (95% confidence interval [CI], 99.65%–99.91%) and sensitivity of 99.5% (97.0%–100%) on samples >14 days after polymerase chain reaction confirmation. Results with a cutoff index >1.00 were interpreted as reactive for anti­–SARS-CoV-2 antibodies and were considered presumptive positive for this study.

### Supplemental SARS-CoV-2 Antibody Testing

Presumptive-positive samples were further tested on the cobas e601 analyzer using the Elecsys Anti-SARS-CoV-2 S ECLIA (Roche Diagnostics; reference 09289267190), a qualitative and semiquantitative assay detecting S subunit 1 (S1) antibodies. The assay predominantly captures immunoglobulin G (IgG) with a positive agreement of 96.6% (95% CI, 93.35%–98.51%) for samples collected >15 days after a positive polymerase chain reaction result and a specificity of 100% (99.7%–100.0%). Qualitative results are reported as the analyte concentration in units per milliliter. Results ≥0.80 to ≤250 U/mL were interpreted as anti–SARS-CoV-2 S reactive and results <0.80 U/mL as negative. Samples >250 U/mL (the upper limit of the assay) were reflexed to serial 1:2 dilution testing until obtainment of a value within the assay's dynamic range.

Presumptive-positive samples were tested on the LABScreen COVID Plus assay (One Lambda; www.onelambda.com) at the ARC Histocompatibility Laboratory Services. The LABScreen COVID Plus is a flow cytometric, qualitative antibody detection assay designed to measure SARS-CoV-2 IgG antibodies. This assay uses microbeads coated with purified SARS-CoV-2 antigens to detect antibodies to 5 SARS-CoV-2 targets (extracellular S domain, S1, S subunit 2 [S2], receptor-binding domain [RBD] and N proteins) and the S1 fragment of 4 seasonal human cold coronaviruses (human coronavirus [HCoV] 229E, HCoV HKU1, HCoV NL63, and HCoV OC43), SARS-CoV, and MERS-CoV. Reactive results were assigned to any target-specific bead regions demonstrating a higher baseline value than the cutoff, using a lot-specific worksheet.

Presumptive-positive samples identified by the Elecsys Anti-SARS-CoV-2 Total N ECLIA were sent to Creative Testing Solutions to be tested using the VITROS Anti-SARS-CoV-2 N chemiluminescent immunoassay (ChLIA; Ortho-Clinical Diagnostics; reference 6199975) and the VITROS Anti-SARS-CoV-2 S1 ChLIA (reference 6199922). With plasma samples collected >15 days after symptom onset, the VITROS Anti-SARS-CoV-2 N assay, a qualitative ChLIA, detects total N SARS-CoV-2 antibodies with a positive agreement of 97.8% (95% CI, 88.4%–99.6%) and a negative agreement of 99.2% (97.9%–99.7%). With plasma samples collected >8 days after symptom onset, the VITROS Anti-SARS-CoV-2 S1 assay, a qualitative ChLIA, detects total SARS-CoV-2 S antibodies with a positive agreement of 100% (95% CI, 92.7%–100.0%) and a clinical sensitivity of 100% (99.1%–100.0%). With either ChLIA, results with a signal-to-cutoff ratio >1.00 are considered reactive.

### Confirmatory SARS-CoV-2 Antibody Testing

Presumptive-positive sample confirmatory testing was done at Emory University (Atlanta, Georgia) using an experimental anti­–SARS-CoV-2 S RBD IgG assay (Wuhan-1 strain) [4] (Supplementary Figure 1). Antibodies to SARS-CoV-2 RBD were detected as described elsewhere [4, 5] (Supplementary Methods). Presumptive-positive samples having an end-point titer ≥200 U/mL were considered confirmed.

### Neutralization Antibody Testing

Confirmed-positive samples were tested at Emory University using an in-house blockade of angiotensin-converting enzyme 2 binding assay (BoAB) to assess the presence of SARS-CoV-2 S (Wuhan and Delta strains) neutralizing antibodies as a specific S-antibody marker and to distinguish between viral strains [6] (Supplementary Methods). The neutralization percentage was calculated based on fluorescence unit inhibition levels for biotinylated angiotensin-converting enzyme 2 and substrate interactions; >20% inhibition was considered neutralized.

### Statistical Analyses

For demographic data, differences in proportions were determined using χ^2^ tests for categorical variables; odds ratio and 95% CIs were calculated for comparisons between all cohorts. Correlation between assays was measured using Pearson correlation analysis and linear regression. Group comparisons were performed using Wilcoxon rank sum tests to assess differences between 2 sample sets (ie, those with Wuhan vs Delta neutralizing activity or specific assay reactivity for samples collected from 13 December 2019 to 22 March 2020 versus 23 March to 5 July 2020). Values were considered significant at *P* ≤ .05. Analyses were performed using SAS software (version 9.4; SAS Institute).

## RESULTS

### Evaluation of the Presence of Antibodies Against SARS-CoV-2

A repository of 46 120 routine blood donation samples was created from donors residing in 46 states plus the District of Columbia (in contrast to the earlier study [3], including 7389 samples from donors residing in 9 states in the Northeast, the upper Midwest, and the West coast). All except 5 samples of the 7389 were retested as part of this study.

Of 46 120 unique donation samples tested by the N-specific Elecsys Anti-SARS-CoV-2 ECLIA, 92 (0.2%) were reactive, with a mean cutoff index of 23.60 (range, 1.03–174.30); these 92 samples were considered presumptive positive, of which 91 had volume for further testing. Multiple assays (anti-N or anti-S directed) were used to increase the robustness of providing a SARS-CoV-2 interpretation, whether confirmed positive or not; accordingly, the selection of assays includes the use of an S-specific neutralization assay for both Wuhan and Delta strains.

**Figure 1.**
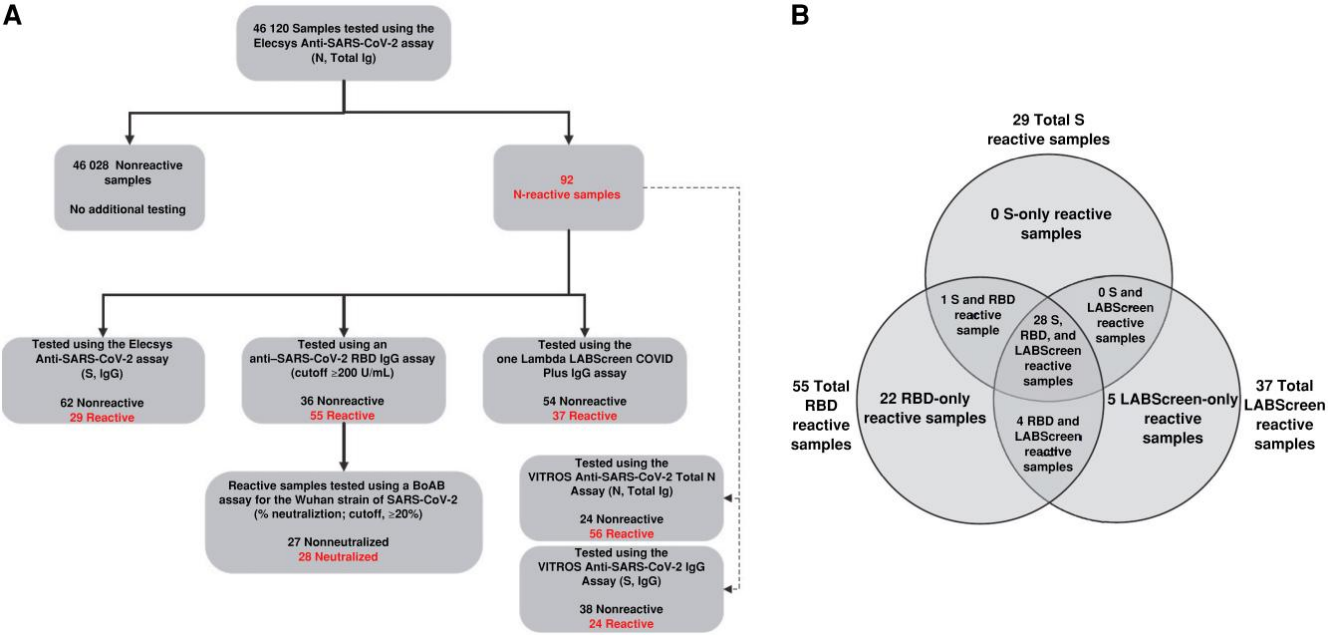
*A*, Study testing algorithm and sample flow. Donation samples (n = 46 120) were tested using the Roche Elecsys Anti-SARS-CoV-2 electrochemiluminescence assay (ECLIA) detecting nucleocapsid (N) and total immunoglobulins (Total Ig). The 92 anti-N­–reactive samples (referred to as *presumptive positives*) with adequate volume (n = 91) were tested using the semiquantitative Elecsys Anti-SARS-CoV-2 S immunoglobulin G (IgG) ECLIA, a research semiquantitative anti–severe acute respiratory syndrome coronavirus (SARS-CoV-2) receptor-binding domain (RBD) IgG assay, and the One Lambda LABScreen COVID Plus IgG assay. The 55 RBD reactive samples were considered confirmed positive and were further tested using a research pseudoneutralization assay, the blockade of angiotensin-converting enzyme 2 binding (BoAB) assay (directed to the Wuhan strain). The 91 presumptive positives with adequate volume were further investigated using the Ortho VITROS Anti-SARS-CoV-2 Total Ig N and VITROS Anti-SARS-CoV-2 Total Ig S chemiluminescent immunoassays (ChLIAs), noted by dashed lines in the testing flow. Of the Elecsys anti-N ECLIA–reactive samples, 1 had insufficent volume for further testing (QNS) after initial screening, 11 were QNS with the VITROS Anti-SARS-CoV-2 Total Ig N assay, and 29 were QNS with the VITROS Anti-SARS-CoV-2 Total Ig S ChLIA. *B,* Results of antibody testing of 91 presumptive-positive samples with further anti–SARS-CoV-2 assays. Each of the 91 Elecsys anti-N ECLIA reactive samples was tested using Elecsys anti-S ECLIA and research anti-RBD assays as well as the LABScreen COVID Plus assay, as shown in *A*. Reactivity to the anti-S and anti-RBD assays established criteria (manufacturer's insert or internal validation) for the cutoff, with reactivity read as units per milliliter, whereas response to any of the 5 anti–SARS-CoV-2 targets of the LABScreen assay was defined as reactive if above the manufacturer's established cutoff. Of the 91 total anti-N-ECLIA reactive samples, 29 (31.9%) were anti-S ECLIA reactive in combination with other markers, 55 (60.4%) anti-RBD reactive alone or in combination with other markers, and 37 (40.7%) LABScreen assay reactive alone or in combination with other markers; 28 (30.8%) were reactive by all 3 assays (anti-S ECLIA, anti-RBD and LABScreen). Dual-marker reactivity included 1 (1.1%) by anti-S and anti-RBD assay and 4 (4.4%) by anti-RBD and LABScreen assay. Single-marker reactivity included 22 (24.2%) by anti-RBD assay only and 5 (5.5%) by LABScreen assay only.

Of the 91 samples that underwent further testing, 29 (31.9%) had anti-S ECLIA reactivity including a subset of 24 (82.8%) with Wuhan neutralizing activity, while 55 (60.4%), including all anti-S–reactive samples, were anti-RBD reactive and considered confirmed positive (Figure 1*A*, solid line, and Table 1). Of the 55 anti-RBD confirmed positives, 29 (52.7%) yielded neutralization-positive results (BoAB directed to the Wuhan strain only [n = 13] or Wuhan in combination with Delta [n = 15], with 1 additional sample neutralizing only Delta; Table 1). The single sample with Delta-only neutralizing activity without Wuhan was collected at a later date in the pandemic (24 April 2020). The 15 samples having dual neutralizing reactivity had significantly higher neutralization percentages for Wuhan (as the primary circulating strain) than for Delta (*P* = .03; 53.96% vs 37.19%, respectively); Delta neutralization reactivity likely indicates cross-reactivity.

**Table 1.** Number and Percentage of Reactive Samples by Testing Assays

Testing Assay	Samples, No. (%)		
	Presumptive-Positive Samples (n = 91)	Confirmed-Positive Samples (n = 55)	BoAB for Wuhan Strain Neutralized Samples (n = 28)
Elecsys Anti-SARS-CoV-2 ECLIAs (Roche)			
Anti-N	91 (100)	55 (100)	28 (100)
Anti-S	29 (31.9)	29 (52.7)	24 (85.7)
Anti–SARS-CoV-2 RBD	55 (60.4)	55 (100)	28 (100)
BoAB			
Wuhan only	NA	13 (23.6)	13 (46.4)
Delta only	NA	1 (1.8)	0 (0.0)
Wuhan and Delta	NA	15 (27.3)	15 (53.6)
LABScreen COVID Plus assay			
SARS-CoV-2 S	30 (33)	29 (52.7)	24 (85.7)
SARS-CoV-2 S1	27 (29.7)	27 (49.1)	24 (85.7)
SARS-CoV-2 S RBD	28 (30.8)	28 (50.9)	24 (85.7)
SARS-CoV-2 S2	24 (26.4)	22 (40)	20 (71.4)
SARS-CoV-2 N	30 (33)	27 (49.1)	21 (75)
>1 Analyte reactive^a^	37 (40.7)	32 (58.2)	24 (85.7)
>2 Analytes reactive	29 (31.9)	29 (52.7)	24 (85.7)
>3 Analytes reactive	27 (29.7)	27 (49.1)	24 (85.7)
>4 Analytes reactive	24 (26.4)	24 (43.6)	22 (78.6)
All 5 Analytes reactive	21 (23.1)	21 (38.2)	19 (67.9)

Abbreviation: BoAB, blockade of angiotensin-converting enzyme 2 binding; ECLIA, electrochemiluminescence assay; N, nucleocapsid; NA, not available; RBD, receptor-binding domain; S, spike; S1, S subunit 1; S2, S subunit 2; SARS-CoV-2, severe acute respiratory syndrome coronavirus 2.

^a^Reactivity to >1 analyte equates to overall sample reactivity.

The mean anti-RBD IgG end-point titer for the 55 confirmed-positive samples was 6259.53 U/mL (range, 213.88–123 056.85 U/mL). The mean anti-RBD IgG end-point titer for the 29 anti-S–reactive subset was 7831.35 U/mL (range, 459.96 to 123 056.85 U/mL). The mean IgG end points for both the anti-RBD confirmed positives and the anti-S–reactive subset were similar; each had a wide quantitative range of end points reflecting variability over the duration of study with increases in reactivity over time (as shown in subsequent analyses). Additional supplemental testing using the multiplex, semiquantitative LABScreen IgG assay yielded 37 of 91 presumptive-positive samples (40.7%) reactive to ≥1 SARS-CoV-2 analytes (Figure 1*A*, solid line, and Table 1).

Figure 1 *B* shows the 91 anti-N ECLIA-reactive samples having further reactivity: 29 (31.9%) anti-S ECLIA reactive in combination with other markers/assays, 55 (60.4%) anti-RBD reactive alone or in combination with other markers/assays, 37 (40.7%) LABScreen reactive alone or in combination with other markers/assays, and 28 (30.8%) reactive for all 3 markers/assays. Dual marker reactivity included 1 (1.1%) by anti-S and anti-RBD and 4 (4.4%) by anti-RBD and LABScreen. Single marker reactivity included 22 (24.2%) by anti-RBD only and 5 (5.5%) by LABScreen only (these 5 LABScreen-only reactive samples were not considered confirmed since lacking both anti-RBD and anti-S reactivity but with reactivity instead to anti-N targets).

The 91 anti-N ECLIA-reactive samples were also tested using commercial ChLIAs (Ortho VITROS anti-S and anti-N) for comparison with the commercial Roche ECLIAs. Of the subset of samples available for testing using ChLIAs, 24 of 62 (39%) and 56 of 80 (70%) were ChLIA-reactive to anti-S total immunoglobulin and anti-N IgG, respectively (Figure 1*A*; dotted line), with 90% and 96% neutralized, respectively (Supplementary Tables 1 and 2). The signal strengths of the 2 commercial anti-S assays (ChLIA vs ECLIA) were highly correlated (Supplementary Figure 2*A*–2*C*) for the available anti-N ECLIA-reactive samples (62 of 91), for the anti-RBD confirmed-positive samples (40 of 55), and for the neutralization-positive samples (20 of 28 Wuhan) (all *P* < .001; *R*^2^ between 92% and 94%). Similarly, the 2 commercial anti-N assays were highly correlated (Supplementary Figure 3*A*–3*C*) for the available anti-N ECLIA-reactive samples (80 of 91), for the anti-RBD confirmed-positive samples (50 of 55), and for the neutralization-positive samples (27 of 28 Wuhan) (all *P* < .001; *R*^2^ between 45% and 68%). Using several reactive sample sets (anti-N, anti-RBD, and neutralization), each with a high positive correlation, confirms the agreement between the 2 commercial sets of assays.

**Figure 2.**
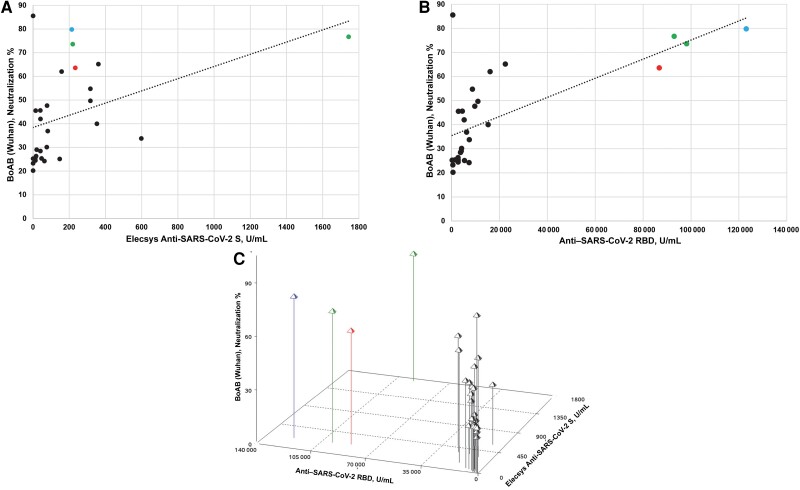
Anti–severe acute respiratory syndrome coronavirus 2 (SARS-CoV-2) antibody level comparisons for neutralized-positive samples. *A*, Correlation between the Elecsys Anti-SARS-CoV-2 S (spike) electrochemiluminescence assay (ECLIA) and the blockade of angiotensin-converting enzyme 2 (BoAB) assay for the 24 anti-S–reactive, confirmed-positive samples with neutralization by the BoAB assay (directed to the Wuhan strain; *P* = .02; *R* ^2^ = 19.5%). *B*, Correlation between the anti­–SARS-CoV-2 receptor-binding domain (RBD) assay and the BoAB assay (Wuhan) for the 28 neutralized-positive samples (*P* < .001, *R* ^2^ = 47.5%). *C*, Three-dimensional comparison of results for the 28 neutralized-positive samples for reactivity to anti-RBD and anti-S (in units per milliliter) and the neutralization percentage. Each line represents a single sample. The x-axis shows the sample titers determined by the anti-RBD assay (*B*; 28 of 28 reactive); the y-axis, the sample titers as determined by the anti-S assay (*A*; 24 of 28 reactive); and the z-axis, the neutralization percentage obtained by the BoAB assay (Wuhan; n = 28). Collection dates for 4 samples with the highest reactivity observed by a combination of anti-S, anti-RBD, and/or the greatest neutralization activity are 23 March, 18 and 23 April, and 1 May 2020; in all panels these samples are highlighted by month of collection: March (*red*), April (*green*), and May (*blue*).

For the 28 Wuhan neutralization-positive samples, the relationships between neutralizing activity (BoAB) and both anti-S ECLIA reactivity (*P* = .02; *R*^2^ = 19.5%; n = 24) and anti-RBD reactivity (*P* < .001; *R*^2^ = 47.5%; n = 28) were significant (Figures 2*A*, *B*). The reactivity of the 3 assays was also directly compared, highlighting that the positive relationships observed were driven primarily by 4 samples collected between 23 March and 1 May 2020, each with high anti-RBD end-point titers, relatively high anti-S values, and the greatest neutralization activity (Figure 2*C*). This agreement was expected, as the RBD is located within the S protein and neutralization is based on the S protein.

Most of the 91 anti-N ECLIA-reactive (presumptive positives) were from repeat donors from the Southern United States, who were 18–24 or 40–54 years old and who donated from 14 April to 24 May 2020 and from 25 May to 5 July 2020 (0.76% and 0.58% reactive for those periods, respectively; Supplementary Table 3). Among the 55 anti-RBD confirmed-positive donors, again, most were 18–24-year-old repeat donors from the Southern United States who donated between 14 April and 5 July 2020 (0.70%–0.47%); 5 July 2020 was the last collection date. When compared with a reference group, each attribute was significant (*P* < .05 for age, donation status, sample collection date, and US census region). The only demographic attribute that was not significant was race/ethnicity, for the comparison between white/non-Hispanic and other race/ethnicities.

**Figure 3.**
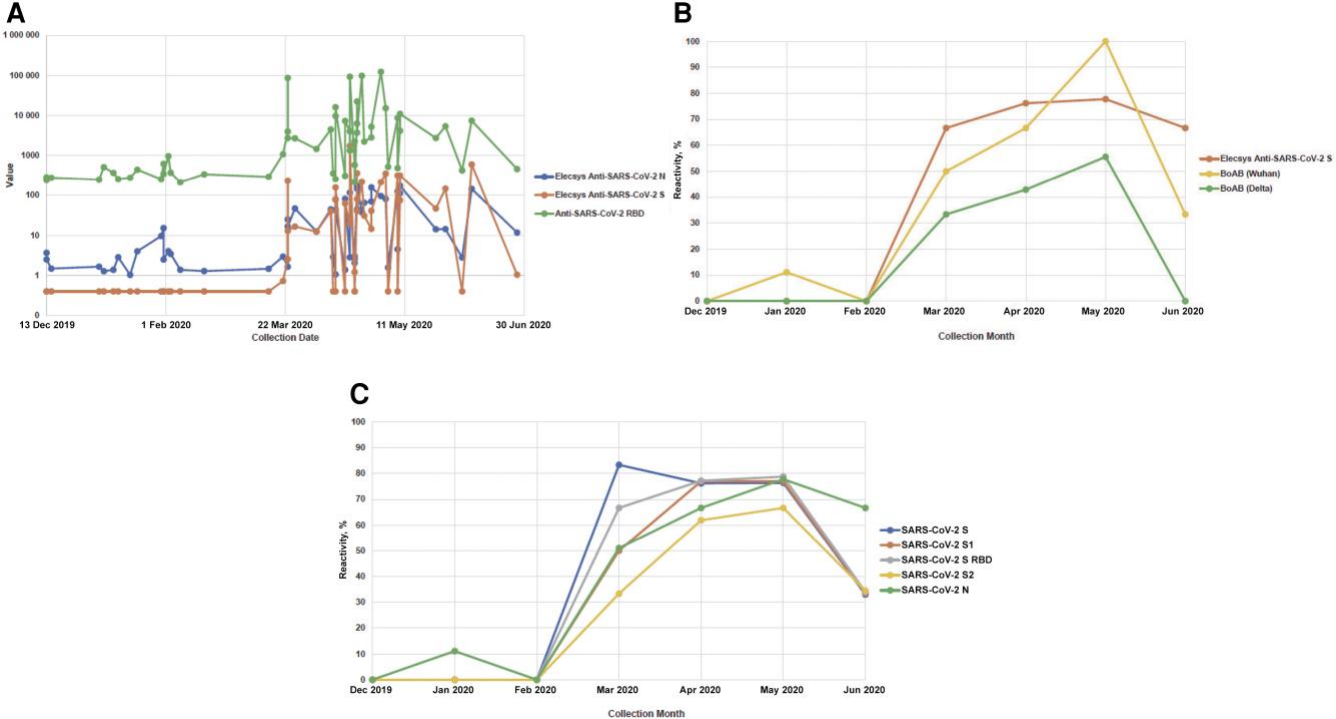
Reactivity of severe acute respiratory syndrome coronavirus 2 (SARS-CoV-2) confirmed-positive samples over time. Reactivity over time where all 3 panels (*A, B,* and *C*) show an upward trend in raw values/percentage reactivity for the 55 confirmed-positive samples (collected from 13 December 2019 to 5 July 2020) correlating with the start of the pandemic in the United States. *A*, Values for samples over time with each assay represented by a separate line: Elecsys Anti-SARS-CoV-2 N (nucleocapsid) electrochemiluminescence assay (ECLIA) (reactivity based on cutoff index), Elecsys Anti-SARS-CoV-2 S (spike) ECLIA (reactivity measured in units per milliliter) and anti–SARS-CoV-2 receptor-binding domain (RBD) (units per milliliter). All samples were reactive for anti-N and anti-RBD; anti-S­–tested samples with >0.80 U/mL were considered reactive. Reactivity by assay was compared for samples collected over 2 periods of time: 13 December 2019 to 22 March 2020 versus 23 March to 5 July 2020; for each assay (anti-N, anti-RBD, and anti-S), reactivity in the latter period was significantly higher (*P* < .001). *B*, Percentage reactivity over time for the anti-S (units per milliliter) and the blockade of angiotensin-converting enzyme 2 binding (BoAB; neutralization percentage for either Wuhan or Delta variant) assays. *C*, Percentage reactivity over time for each of the 5 SARS-CoV-2 analytes of the LABScreen COVID Plus assay; each line indicates an individual analyte. Abbreviations: S1, S subunit 1; S2, S subunit 2.

Far fewer confirmed-positive samples, as well as those having lower signal levels across all assays, were observed between 13 December 2019 and 3 March 2020 (Figure 3*A*–3*C* and Supplementary Table 3). Of the 91 presumptive positives, only 34 (37%) anti-N ECLIA-reactive and 16 (29%) of 55 anti-RBD confirmed-positive samples were identified from 13 December 2019 to 3 March 2020, peaking to 57 of 91 and 39 of 55 (63%–71%), respectively, by 5 July 2020. Confirmed-positive rates increased from 0.07%–0.06% to a peak of 0.70%–0.47% over these 2 time periods (Supplementary Table 3). Figure 3*A* follows the 55 anti-RBD confirmed-positive samples with respect to their ECLIA (anti-N and anti-S) reactivity as well as their anti-RBD reactivity, demonstrating a surge in reactivity starting in March 2020 (*P* < .001 for the difference in reactivity for each marker for the 2 time periods: 13 December 2019 to 22 March 2020 vs 23 March to 5 July 2020). Of note, only 9 (0.07%) confirmed-positive samples were identified among the 13 364 donations collected between 13 December 2019 and 22 January 2020. In addition, no confirmed-positive samples were anti-S ECLIA reactive until 23 March 2020, again noting low reactivity in the 21 samples listed in Supplementary Table 4.

The geographic distribution of the donors of the 21 confirmed-positive samples (anti-N and anti-RBD) collected from 13 December 2019 to 23 March 2020 was similar to the distribution for the repository samples; of the 21 donors, 43% resided in the Midwest, 38% in the Northeast and 19% in the Western United States, suggesting that SARS-CoV-2 had already spread across the United States. Similarly, starting in March 2020, increasing trends were observed for neutralizing activity (Wuhan and Delta) (Figure 3*B*) and for the 5 SARS-CoV-2 targets of the LABScreen assay (S, S1, S RBD, S2, and N) (Figure 3*C*).

### Evaluation of the Presence of Antibodies Against Other Coronaviruses

**Figure 4.**
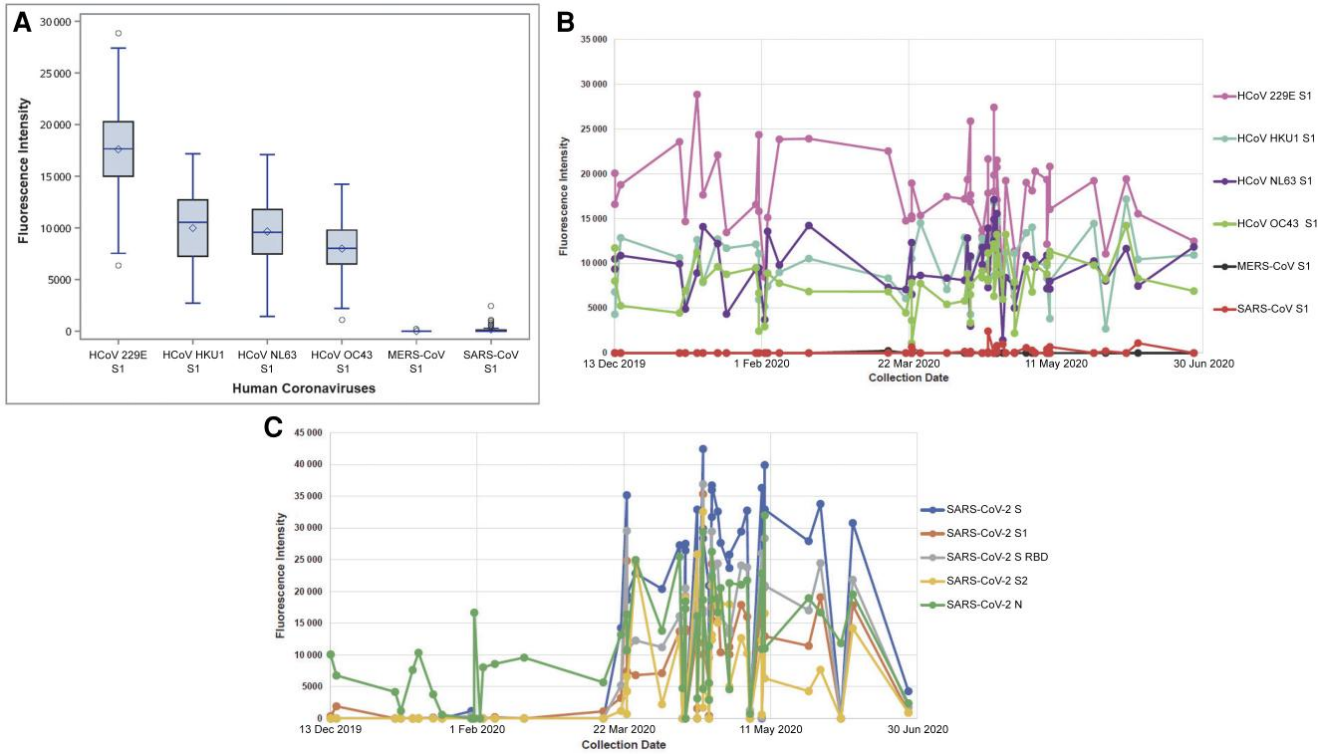
Evaluation of the presence of antibodies against other coronaviruses. The presence of antibodies against the 4 common cold coronaviruses (human coronavirus [HCoV] 229E, HCoV HKU1, HCoV NL63, and HCoV OC43), Middle East respiratory syndrome coronavirus (MERS-CoV), and severe acute respiratory syndrome (SARS) coronavirus (SARS-CoV), for the 55 SARS coronavirus 2 (SARS-CoV-2) confirmed-positive samples was assessed with the LABScreen COVID Plus IgG assay. *A*, Box plots of the LABScreen COVID Plus assay showing the fluorescence intensity for the 4 common cold coronaviruses, MERS-CoV, and SARS-CoV. All 55 samples showed some to minimal reactivity to the spike (S) subunit 1 (S1) of the 4 common cold coronaviruses but showed virtually no reactivity to S1 of MERS-CoV or SARS-CoV. *B*, Fluorescence intensities for the 55 SARS-CoV-2 confirmed-positive samples to each common cold coronavirus, MERS-CoV, and SARS-CoV by the collection date. The S reactivity for each cold coronavirus was compared for samples collected over 2 periods of time: 13 December 2019 to 22 March 2020 versus 23 March to 5 July 2020. No significant trends in fluorescence intensities were observed over time for any of these viruses; MERS-CoV and SARS-CoV were excluded since minimal or no reactivity was observed. *C*, Fluorescence intensities of the 55 SARS-CoV-2 confirmed-positive samples to each LABScreen COVID Plus SARS-CoV-2 analyte show the identical trends observed in Figure 3*B*. That is, reactivity by assay were compared for samples collected over 2 periods: 13 December 2019 to 22 March 2020 versus 23 March to 5 July 2020; for each assay (anti-S, S1, and subunit 2 [S2], nucleocapsid [N] and receptor-binding domain [RBD]), the latter time period had significantly higher reactivity (*P* < .001).

The presence of antibodies against the 4 common human cold coronaviruses (HCoV 229E, HCoV HKU1, HCoV NL63, and HCoV OC43), MERS-CoV, and SARS-CoV for the 55 SARS-CoV-2 anti-RBD confirmed-positive samples was assessed. Box plots show the fluorescence intensity for these other coronaviruses (Figure 4*A*). All 55 SARS-CoV-2 confirmed-positive samples evaluated had some to minimal antibody reactivity to the S1 of the 4 common cold coronaviruses but showed virtually no antibody reactivity to the S1 of MERS-CoV or SARS-CoV. The fluorescence intensities of these 55 samples to each common cold coronavirus, MERS-CoV, and SARS-CoV are shown in Figure 4*A* in aggregate and in Figure 4*B* over time.

No significant trends In fluorescence intensities for any of the common cold coronaviruses were observed over time (when samples collected from 13 December 2019 to 22 March 2020 were compared with those collected on or after 23 March 2020), indicating constant viral exposure in the population to these coronaviruses in contrast to SARS-CoV-2. In addition, no notable reactivity occurred for MERS-CoV or SARS-CoV. Fluorescence intensities of the 55 SARS-CoV-2 confirmed-positive samples for each LABScreen SARS-CoV-2 analyte show the identical trends previously observed (Figure 4*C* compared with Figure 3*A*, with both figures showing significant increases in SARS-CoV-2 reactivity after 22 March 2020; *P* < .001). The fluorescence intensity values of SARS-CoV-2 negative control samples were 0 for the SARS-CoV-2 analytes (data not shown) but comparable to the values shown in Figure 4*B* for the 4 common cold coronaviruses.

## DISCUSSION

The time of introduction of SARS-CoV-2 into the United States has not been definitively established [1, 2]. In Wuhan, Hubei Province, China, the first cluster of clinical cases later attributed to SARS-CoV-2 was identified on 7 January 2020 [7]. The first RNA-positive asymptomatic blood donor was identified in Wuhan on 28 January 2020; by 4 March, 4 infected blood donors had been identified (albeit all antibody nonreactive attributed to early infection) [8]. In the United States, as of 4 February 2020, 11 COVID-19 clinical cases were reported to the Centers for Disease Control and Prevention [9]. In a previous US study of approximately 7400 routine blood donation samples from healthy donors collected from 13 December 2019 to 19 January 2020, using a research-based, full-length anti-S assay, 1.4% antibody reactivity was identified across 9 states [3]. Most reactive samples in that study had reactivity to ≥1 other supplemental assay used (>93% neutralized, while 44% had S1-specific reactivity; S1 has been reported to be more specific for SARS-CoV-2 than total S) [10]. This suggests that SARS-CoV-2 was likely introduced into the United States before 19 January 2020, the date of the first reported clinical cases [1, 2].

In this follow-up study, the entire repository of 46 120 samples covering donors who resided in the continental United States was tested with the goal of identifying the earliest SARS-CoV-2 antibody (anti-N) responses. Although samples from the entire United States were represented, the repository samples were concentrated in the same 9 states in the Northeast, Midwest, and Western United States included in the previous study [3]. Since there is no accepted reference standard antibody assay to establish infection, multiple anti-N and anti-S assays were used to demonstrate that the observed reactivity of these early anti-N–reactive samples could be conclusively attributed to SARS-CoV-2 infection. The secondary goals were to monitor increases in serologic reactivity over the first 6 months of the COVID-19 pandemic and to assess patterns of reactivity of anti-N–reactive/anti-RBD confirmed-positive samples to the common cold coronaviruses, as well as to SARS-CoV and MERS-CoV.

Pan-immunoglobulin anti-N testing using the same commercial SARS-CoV-2 assay was reported to be robust, with few false-positives [11, 12]. As reported in studies of seroprevalence early in the COVID-19 pandemic, 2 assays were required for confirmed positivity (anti-N and anti-RBD) [12]. In our study, we found a 0.2% anti-N seroprevalence, far lower than the 1.4% in the earlier US study using a subset of the repository, likely due to the larger sample size (46 120 vs 7389) from a larger geographic area. Results of further testing of the available 91 anti-N–reactive samples demonstrated reactivity that ranged from 60.4% with a research-based anti-RBD assay to 40.7% with the commercial LABScreen assay and 31.9% with a commercial anti-S assay (ECLIA); finally, 50.9% (28 of 55) of the anti-RBD confirmed-positive samples had neutralizing ability demonstrated by a research-based BoAB neutralization assay (Table 1).

Neutralization was included as a highly specific marker of anti-S reactivity and to differentiate SARS-CoV-2 strains. Of note, S1-specific antibody reactivity was similar in both studies when commercial assays were compared (44% for anti-S ChLIA in the previous study [3] compared with 39% for anti-S ChLIA and 35% for anti-S ECLIA in the current study; Supplementary Table 1). Anti-N reactivity was also similar between the 2 commercial assays (ie, 70% of the ECLIA anti-N–reactive samples were reactive by anti-N ChLIA; Supplementary Table 2). In addition, the signal strengths for both anti-N and, particularly, anti-S, commercial assays were highly correlated (*P* < .001; Supplementary Figures 2 and 3).

Most SARS-CoV-2 presumptive-positive and confirmed-positive donors were 18–24-year-old repeat donors from the Southern United States who donated between 14 April and 5 July 2020, consistent with early clinical cases, as the COVID-19 pandemic spread throughout the United States. Presumptive-positive and confirmed-reactive rates increased from 0.10%–0.17% to 0.58%–0.76% over the 2 time periods (13 December 2019 to 3 March 2020 and then through 5 July 2020; Supplementary Table 3); parallel patterns were observed for all SARS-CoV-2 markers tested (Figures 3*A*–3*C* and 4*C*), indicating that low levels of SARS-CoV-2 likely existed in the United States starting as early as 13 December 2019.

Anti-S ECLIA reactivity was absent in the earliest confirmed-positive samples; in addition, we saw the same absence of reactivity with the neutralization and the LABScreen assays; however, all of these anti-N–reactive samples were confirmed by anti-RBD assay. One interpretation of this finding is that we preselected (enriched) for anti-N reactivity by the use of the anti-N assay, and while these confirmed by the anti-RBD assay (a subset of anti-S), no anti-S reactivity was observed by multiple assays until 23 March 2020 (Supplementary Table 4). In addition, the detection of anti-N antibodies in total immunoglobulin assays is more sensitive than the detection of anti-S antibodies, as the former may appear earlier due to accumulating N early in infection [13–17]. Moreover, samples having less than full antigenic responses may be the result of infections observed early in the pandemic and may thus represent recent infection.

In contrast to patterns observed for antibody markers to SARS-CoV-2, which paralleled the COVID-19 pandemic, no increasing trends in reactivity to anti-S or anti-N markers for any of the common cold coronaviruses were observed over time, indicating constant viral exposure in the population. In addition, no notable reactivity occurred for MERS-CoV or SARS-CoV. The history and interrelationships between all of these coronaviruses is reviewed elsewhere [18]. While it is possible that preexisting antibodies to other coronaviruses did cross-react with our SARS-CoV-2 confirmed-positive samples, they did not influence the patterns of reactivity to the SARS-CoV-2 analytes observed over time. This is also driven by the use of an anti-N test for primary SARS-CoV-2 antibody screening, which demonstrates lesser cross-reactivity with other coronaviruses than do anti-S analytes [3].

Limitations of the current study include that in neither study (Basavaraju et al [3] or the current study) were all samples tested simultaneously using assays to detect both anti-N and anti-S for a direct comparison, nor were molecular tests used. However, in both studies of samples broadly representative of the United States from asymptomatic individuals who had successfully donated blood, the general conclusion is that the rates of SARS-CoV-2 antibodies were low but present very early in the pandemic, likely indicating that infections occurred in the United States before clinical cases. Finally, in the current study, we showed that SARS-CoV-2 antibodies were unrelated to any of the 4 common human cold coronaviruses, whose rates did not vary over the study period.

## Supplementary Data

Supplementary materials are available at *Open Forum Infectious Diseases* online. Consisting of data provided by the authors to benefit the reader, the posted materials are not copyedited and are the sole responsibility of the authors, so questions or comments should be addressed to the corresponding author.

## Supplementary Material

ofae351_Supplementary_Data
