# Supplementary material for: Serologic Testing of US Blood Donations to Identify Severe Acute Respiratory Syndrome Coronavirus 2 and Other Coronaviruses, December 2019 to July 2020
Source: Open Forum Infect Dis. 2024 Jun 28;11(7):ofae351. doi: 10.1093/ofid/ofae351 (PMC11257073; doi:10.1093/ofid/ofae351)
Supplement: ofae351_Supplementary_Data [file ofae351_supplementary_data.zip › Supplement COVID-19 Stramer et al OFID- jk CLEAN.docx]

**Serologic Testing of US Blood Donations to Identify Severe Acute Respiratory Syndrome Coronavirus-2 and other Coronaviruses, December 2019-July 2020**

Kacie Grimm^1^, Paula Saá PhD^1^, Narayanaiah Cheedarla^2^, Michael S. Gerty^3^, Jamel A. Groves MS^1^, Roger Y. Dodd PhD^1^, John Roback MD^2^, Susan L. Stramer, PhD MS^4^

^1^Scientific Affairs, American Red Cross, Rockville MD

^2^Department of Pathology and Laboratory Medicine, Emory University School of Medicine, Atlanta GA

^3^Histocompatibility Laboratory, American Red Cross, Philadelphia PA

^4^Infectious Disease Consultant, North Potomac, MD

**Supplemental Appendix**

**Table on Contents**

**Page**

**Supplemental Methods 3**

**Confirmatory SARS-CoV-2 Antibody Testing 3**

**Neutralization Antibody Testing 4**

**ACE-2 Biotinylation 4**

**Spike conjugation with magnetic beads 5**

**References 6**

**Supplemental Table 1. 7**

**Supplemental Table 2. 8**

**Supplemental Table 3. 9**

**Supplemental Table 4. 11**

**Supplemental Figure 1. 13**

**Supplemental Figure 2. 13**

**Supplemental Figure 3. 14**

**Supplemental Methods:**

*Confirmatory SARS-CoV-2 Antibody Testing*

Confirmatory testing of presumptive-positive samples was done at Emory University (Atlanta, GA) using an experimental anti-SARS-CoV-2 S RBD IgG assay.^1^ The RBD of the Wuhan 1 strain of SARS-CoV-2 was produced by transfection using FreeStyle 293-F cells.^2^ Briefly, FreeStyle 293F cells were seeded at a density of 2.0E6 cells/mL in Expi293 expression media and incubated with shaking overnight at 37°C and 127 rpm with 8% CO_2_. The following day, 2.5E6 cells/mL were transfected using ExpiFectamine^TM^ 293 transfection reagent (ThermoFisher) according to the manufacturer protocol. Transfected cells were incubated with orbital shaking for 4-5 days at 37°C, 127 rpm, 8% CO_2_. Supernatants containing secreted trimeric ectodomains were collected by centrifugation at 4,000 x g for 20 min at 4°C. Clarified supernatants were filtered using a 0.22-µm stericup filter (ThermoFisher) and loaded onto pre-equilibrated affinity columns for protein purification. The RBD was purified using His-Pur Ni-NTA resin (ThermoFisher). Briefly, His-Pur Ni-NTA resin was washed twice with phosphate-buffered saline (PBS) by centrifugation at 2000 x g for 10 min. The resin was resuspended with the RBD supernatant and incubated for 2 h on a shaker at room temperature (RT). Next, gravity flow columns were loaded with supernatant-resin mixture and washed (25 mM Imidazole, 6.7 mM NaH2PO4.H_2_O and 300 mM NaCl in PBS) four times, after which proteins were eluted in elution buffer (235 mM Imidazole, 6.7 mM NaH2PO4.H2O and 300 mM NaCl in PBS). Eluted proteins were dialyzed against PBS using Slide-A-lyzer Dialysis Cassette (ThermoScientific) and concentrated using 10 kDa Amicon Centrifugal Filter Unit, at 2000 x g at 4°C. The concentrated protein eluate was run and fractionated on a Sepharose 600 (GE Healthcare) column on an AktaTMPure (GE Healthcare). Fractions corresponding to the molecular weight of each protein were pooled and concentrated as described above. Proteins were quantified by BCA Protein Assay Kit (Pierce) and quality was confirmed by SDS-PAGE (**Supplemental Figure 1**). Detection of SARS CoV-2 RBD antibodies was performed as described.^1^ Briefly, SARS-CoV-2 RBD was coated on Nunc MaxiSorp plates (Invitrogen) at a concentration of 1 μg/mL in 100 μL PBS at 4°C overnight. Plates were blocked for two hours at RT in PBS/0.05% Tween-20/1% BSA (ELISA buffer). Samples were aliquoted and stored at -80°C before use and serially diluted 1:3 in dilution buffer (PBS-1% BSA-0.05% Tween-20) starting at a dilution of 1:50. Each dilution (100 uL) was added and incubated for 90 min at RT; 100 uL of horseradish peroxidase (HRP)-conjugated IgG-specific secondary antibody, diluted 1:5,000 in ELISA buffer, was added and incubated for 60 min at RT. Development was performed using 0.4 mg/mL o-phenylenediamine substrate (Sigma) in 0.05 M phosphate-citrate buffer pH 5.0, supplemented with 0.012% hydrogen peroxide before use. Reactions were stopped with 1 M HCl, and absorbance was measured at 492 nm. Between each step, samples were washed four times with 300 µL of PBS-0.05% Tween-20. Presumptive positive samples that had an endpoint titer of ≥ 200 U/mL were considered confirmed positive (CP) for this study.

*Neutralization Antibody Testing*

Confirmed-positive samples were tested at Emory University using an in-house blockade of angiotensin converting enzyme-2 (ACE-2) binding assay (BoAB) to assess the presence of neutralizing antibodies. ACE-2 detector biotinylation and SARS-CoV-2 spike (Wuhan and Delta strains) bead conjugation was performed according to the Quanterix Homebrew Detection Antibody Biotinylation and Bead Conjugation Protocols.^3^

*ACE-2 Biotinylation*

Briefly, ACE-2 was buffer exchanged using Amicon filtration into Quanterix biotinylation reaction buffer prior to mixing at 1 mg/mL with a 40X challenge ratio of NHS-PEG4-biotin for 30 min at RT. Cleanup of the biotinylated detection reagent was achieved by a further round of Amicon filtration (50kDa cutoff, cat# UFC905024, Vendor: Millipore) following recovery in biotinylation reaction buffer and determination of protein concentration by BCA method (Pierce™ BCA Protein Assay Kit, cat#23227). A final detector concentration of 0.5 µg/mL was used in the assay.

*Spike conjugation with magnetic beads*

Paramagnetic beads were activated after washing with bead conjugation buffer using 9 µg 1-ethyl-3-

[3-dimethylaminopropyl] carbodiimide (EDC) (10 mg/mL) in a final bead volume of 300 µL containing 4.2E8 beads for 30 min at 4°C with rocking. Following activation, beads were washed with bead conjugation buffer and 300 µL cold spike at 0.2 mg/mL followed by incubation at 4°C with rocking for 2h. Beads were washed and blocked for 45 min at RT, followed by a final wash and resuspension in 300 µL bead diluent. Spike capture beads were stored at 4°C until further use. Percentage of neutralization was calculated based on the fluorescence units to quantify inhibition levels of biotinylated ACE-2 and substrate interactions.

**References**

1. Suthar MS, Zimmerman MG, Kauffman RC. Rapid generation of neutralizing antibody responses in COVID-19 patients. Cell Reports Medicine 2020;1(3). PMID: **32835303**. PMCID: [PMC7276302](http://www.ncbi.nlm.nih.gov/pmc/articles/pmc7276302/). doi: [10.1016/j.xcrm.2020.100040](https://doi.org/10.1016%2Fj.xcrm.2020.100040)
2. Nooka AK, Shanmugasundaram U, Cheedarla N, et al. Determinants of neutralizing antibody response after SARS CoV-2 vaccination in patients with myeloma. J Clin Oncol. 2022;40:3057-64. PMID: **35259002.** PMCID: [PMC9462534](http://www.ncbi.nlm.nih.gov/pmc/articles/pmc9462534/). DOI: [10.1200/JCO.21.02257](https://doi.org/10.1200/jco.21.02257)
3. Cheedarla N, Verkerke HP, Potlapalli S, et al. Rapid, high throughput, automated detection of SARS-CoV-2 neutralizing antibodies against Wuhan-WT, delta and omicron BA1, BA2 spike trimers. iScience 2023;26:108256. PMID: **37965140.** PMCID: [PMC10641509](http://www.ncbi.nlm.nih.gov/pmc/articles/pmc10641509/). DOI: [10.1016/j.isci.2023.108256](https://doi.org/10.1016/j.isci.2023.108256)

**Supplemental Tables**

**Supplemental Table 1. Comparison of reactivity of the Elecsys Anti-SARS-CoV-2 N ECLIA (Roche) presumptive-positive samples (anti-N reactive) on the VITROS S ChLIA (Ortho) and Elecsys S ECLIA (Roche).** The 91 presumptive-positive samples identified by the Elecsys Anti-SARS-CoV-2 N ECLIA were tested by the Elecsys Anti-SARS-CoV-2 S ECLIA and the VITROS Anti-SARS-CoV-2 Total S ChLIA. The table shows the number (and percent) anti-N-reactive samples that were Elecsys S ECLIA reactive and VITROS S ChLIA reactive, for samples with available volume (62/91 presumptive-positive samples, 40/55 confirmed-positive samples and 20/28 BoAB neutralized-positive samples). ECLIA: electrochemiluminescence immunoassay; ChLIA: chemiluminescent immunoassay; S: spike; N: nucleocapsid; BoAB: blockade of ACE-2 binding; No: number; ACE-2: angiotensin converting enzyme-2.

|  | **Presumptive Postive Samples, n=62** | **Confirmed Positive Samples, n=40** | **BoAB (Wuhan) Neutralized Samples, n=20** |
| --- | --- | --- | --- |
| **No. Elecsys S Reactive (%)** | 22 (35) | 22 (55) | 17 (85) |
| **No. VITROS S Reactive (%)** | 24 (39) | 24 (60) | 18 (90) |

**Supplemental Table 2. Comparison of reactivity of the Elecsys Anti-SARS-CoV-2 N ECLIA (Roche) presumptive-positive samples (anti-N reactive) on the VITROS ChLIA N (Ortho).** The 91 presumptive-positive samples identified by the Elecsys Anti-SARS-CoV-2 N ECLIA were tested by the VITROS Anti-SARS-CoV-2 Total N ChLIA. The table shows the number (and percent) anti-N-reactive samples that were VITROS Total N ChLIA reactive for samples with available volume (80/91 presumptive-positive samples, 50/55 confirmed-positive samples and 27/28 BoAB neutralized-positive samples). ECLIA: electrochemiluminescence immunoassay; ChLIA: chemiluminescent immunoassay; N: nucleocapsid protein; BoAB: blockade of ACE-2 binding; No: number; ACE-2: angiotensin converting enzyme-2.

|  | **Presumptive Postive Samples, n=80** | **Confirmed Positive Samples, n=50** | **BoAB (Wuhan) Neutralized Samples, n=27** |
| --- | --- | --- | --- |
| **No. VITROS N Reactive (%)** | 56 (70) | 41 (82) | 26 (96) |

**Supplemental Table 3. Donor demographics.** Demographic characteristics of 46,120 Elecsys ECLIA anti-N-tested donation samples. Presumptive positive samples (n=91) were further tested using the Anti-SARS-CoV-2 RBD assay (n=55 considered confirmed positive). Bold text denotes statistical significance within the demographic category. *0.5 was added to each cell for the purpose of calculating Odds Ratios (ORs) since one of the categories had a value of 0.00 †Other includes African American (2/739 reactive; 0 confirmed), Asian (2/765 reactive; 2 confirmed), Hispanic (5/1229 reactive; 1 confirmed), mixed race (1/471 reactive; 0 confirmed), Native American (1/332 reactive; 1 confirmed), and self-described as "Other" or preferred not to respond to the question (0/340 reactive); ‡The range for collect dates is approximately six weeks. ECLIA: electrochemiluminescence immunoassay; CI: confidence intervals; N: nucleocapsid; RBD: receptor-binding domain.

| Characteristics | No. Tested | No. Screened Reactive (%) | OR (95% CI) | p value |  | No. Confirmed Positive (%) | OR (95% CI) | p value |
| --- | --- | --- | --- | --- | --- | --- | --- | --- |
| Sex |  |  |  |  |  |  |  |  |
| Female | 22354 | 41 (0.18) | 1.00 |  |  | 22 (0.10) | 1.00 |  |
| Male | 23766 | 50 (0.21) | 1.15 (0.76-1.73) | 0.514 |  | 33 (0.14) | 1.41 (0.82-2.42) | 0.209 |
| Age* (years) |  |  |  |  |  |  |  |  |
| 16-17 | 1255 | 0 (0.00) | 0.27 (0.02-4.46) | NA |  | 0 (0.00) | 0.42 (0.03-6.94) | NA |
| 18-24 | 3092 | 11 (0.36) | 2.56 (1.30-5.03) | **0.007** |  | 7 (0.23) | 2.56 (1.11-5.92) | **0.035** |
| 25-39 | 8726 | 19 (0.22) | 1.53 (0.87-2.70) | 0.149 |  | 13 (0.15) | 1.63 (0.82-3.25) | 0.177 |
| 40-54 | 11432 | 30 (0.26) | 1.83 (1.11-3.02) | **0.017** |  | 15 (0.13) | 1.43 (0.74-2.77) | 0.303 |
| >55 | 21615 | 31 (0.14) | 1.00 |  |  | 20 (0.09) | 1.00 |  |
| Race/ethnicity |  |  |  |  |  |  |  |  |
| White | 42244 | 80 (0.19) | 1.00 |  |  | 51 (0.12) | 1.00 |  |
| Other† | 3876 | 11 (0.28) | 1.50 (0.80-2.82) | 0.205 |  | 4 (0.10) | 0.85 (0.31-2.37) | 0.762 |
| Donation Status |  |  |  |  |  |  |  |  |
| First-time | 13809 | 12 (0.09) | 1.00 |  |  | 7 (0.05) | 1.00 |  |
| Repeat | 32311 | 79 (0.24) | 2.82 (1.53-5.17) | **<0.0001** |  | 48 (0.15) | 2.94 (1.33-6.49) | **0.005** |
| Collect Date Range‡ |  |  |  |  |  |  |  |  |
| December 13, 2019-January 22, 2020 | 13263 | 23 (0.17) | 1.00 |  |  | 9 (0.07) | 1.00 |  |
| January 23, 2020-March 3, 2020 | 11182 | 11 (0.10) | 0.57 (0.28-1.16) | 0.117 |  | 7 (0.06) | 0.92 (0.34-2.48) | 0.872 |
| March 4, 2020-April 13, 2020 | 17522 | 27 (0.15) | 0.89 (0.51-1.55) | 0.677 |  | 12 (0.07) | 1.01 (0.43-2.40) | 0.984 |
| April 14, 2020-May 24, 2020 | 3295 | 25 (0.76) | 4.40 (2.49-7.76) | **<0.0001** |  | 23 (0.70) | 10.35 (4.78-22.38) | **<0.0001** |
| May 25, 2020-July 5, 2020 | 858 | 5 (0.58) | 3.37 (1.28-8.90) | **0.009** |  | 4 (0.47) | 6.90 (2.12-22.45) | **<0.0001** |
| US Census Region |  |  |  |  |  |  |  |  |
| Midwest | 22339 | 29 (0.13) | 1.00 |  |  | 18 (0.08) | 1.00 |  |
| Northeast | 11569 | 18 (0.16) | 1.20 (0.67-2.16) | 0.545 |  | 8 (0.07) | 0.86 (0.37-1.97) | 0.719 |
| South | 7624 | 33 (0.43) | 3.34 (2.03-5.51) | **<0.0001** |  | 24 (0.32) | 3.92 (2.13-7.22) | **<0.0001** |
| West | 4588 | 11 (0.24) | 1.85 (0.92-3.70) | 0.078 |  | 5 (0.11) | 1.35 (0.50-3.65) | 0.548 |
| Total | 46120 | 91 (0.20) |  |  |  | 55 (0.12) |  |  |

**Supplemental Table 4. Reactivity of the SARS-CoV-2 confirmed-positive samples from December 19, 2019 to March 23, 2020.** A line listing of reactivity to all tests performed on the complete set of confirmed-positive samples, in addition to state of residence, is provided for the 21 samples having the earliest anti-N reactivity. The tests performed include the Elecsys anti-N ECLIA (COI), Elecsys anti-S ECLIA (U/mL), anti-RBD (U/mL), percent neutralization (% neut) by BoAB (Wuhan and Delta) and the five LABScreen COVID Plus Assay markers for SARS-CoV-2 (anti-S, anti-S1, anti-S RBD, anti-S2, and anti-N). Reactive results are bolded. ECLIA: electrochemiluminescence immunoassay; N: nucleocapsid; COI: cutoff-to-index; S: spike; U/mL; units per milliliter; RBD: receptor-binding domain; BoAB: blockade of ACE-2 binding; ACE-2: angiotensin converting enzyme-2; S1: spike subunit 1; S2: spike subunit 2; neg: negative; pos: positive.

| **Collect Date** | **State of Residence** | **anti-N (COI)** | **anti-S (U/mL)** | **anti-RBD (U/mL)** | **% Neut (Wuhan)** | **% Neut (Delta)** | **LABScreen Spike** | **LABScreen Spike S1** | **LABScreen Spike RBD** | **LABScreen Spike S2** | **LABScreen N** |
| --- | --- | --- | --- | --- | --- | --- | --- | --- | --- | --- | --- |
| 12/13/2019 | California | **3.66** | 0.40 | **242.68** | <20% | <20% | Neg | Neg | Neg | Neg | Neg |
| 12/13/2019 | Oregon | **2.50** | 0.40 | **282.87** | <20% | <20% | Neg | Neg | Neg | Neg | Neg |
| 12/15/2019 | Nevada | **1.48** | 0.40 | **276.77** | <20% | <20% | Neg | Neg | Neg | Neg | Neg |
| 1/4/2020 | Iowa | **1.65** | 0.40 | **247.81** | <20% | <20% | Neg | Neg | Neg | Neg | Neg |
| 1/6/2020 | Wisconsin | **1.27** | 0.40 | **510.60** | <20% | <20% | Neg | Neg | Neg | Neg | Neg |
| 1/10/2020 | Connecticut | **1.38** | 0.40 | **366.51** | <20% | <20% | Neg | Neg | Neg | Neg | Neg |
| 1/12/2020 | Massachusetts | **2.84** | 0.40 | **254.84** | <20% | <20% | Neg | Neg | Neg | Neg | Neg |
| 1/17/2020 | Michigan | **1.03** | 0.40 | **276.31** | <20% | <20% | Neg | Neg | Neg | Neg | Neg |
| 1/20/2020 | Missouri | **4.00** | 0.40 | **441.34** | <20% | <20% | Neg | Neg | Neg | Neg | Neg |
| 1/30/2020 | Massachusetts | **9.62** | 0.40 | **254.00** | **25.27** | <20% | Neg | Neg | Neg | Neg | Neg |
| 1/31/2020 | Maine | **2.49** | 0.40 | **344.65** | <20% | <20% | Neg | Neg | Neg | Neg | **Pos** |
| 1/31/2020 | Missouri | **15.19** | 0.40 | **617.41** | <20% | <20% | Neg | Neg | Neg | Neg | Neg |
| 2/2/2020 | Massachusetts | **4.04** | 0.40 | **960.33** | <20% | <20% | Neg | Neg | Neg | Neg | Neg |
| 2/3/2020 | Connecticut | **3.44** | 0.40 | **370.91** | <20% | <20% | Neg | Neg | Neg | Neg | Neg |
| 2/7/2020 | Kentucky | **1.38** | 0.40 | **213.38** | <20% | <20% | Neg | Neg | Neg | Neg | Neg |
| 2/17/2020 | Michigan | **1.28** | 0.40 | **337.42** | <20% | <20% | Neg | Neg | Neg | Neg | Neg |
| 3/15/2020 | Missouri | **1.46** | 0.40 | **291.08** | <20% | <20% | Neg | Neg | Neg | Neg | Neg |
| 3/21/2020 | Kentucky | **2.94** | 0.73 | **1074.71** | <20% | <20% | **Pos** | Neg | Neg | Neg | **Pos** |
| 3/23/2020 | California | **24.98** | **233.30** | **86762.06** | **63.59** | **25.74** | **Pos** | **Pos** | **Pos** | **Pos** | **Pos** |
| 3/23/2020 | Connecticut | **16.64** | **12.91** | **2726.35** | **24.50** | **32.82** | **Pos** | **Pos** | **Pos** | Neg | Neg |
| 3/23/2020 | Connecticut | **1.65** | **2.54** | **3978.10** | <20% | <20% | **Pos** | Neg | **Pos** | Neg | Neg |

**Supplemental Figure Legends**

**Supplemental Figure 1. Results of SARS-CoV-2 RBD purification.** SARS-CoV-2 RBD protein purification by affinity column chromatography and confirmation by SDS-PAGE. SARS-CoV-2 RBD (Wuhan-1) protein was produced by transfection in FreeStyle 293-F cells of the MN908947 plasmid for Wuhan-1, purified by affinity column followed by size exclusion chromatography, protein concentrated, and then quantified by BCA Protein Assay Kit (Pierce). Quality was confirmed by SDS-PAGE (as shown).

**Supplemental Figure 2. Anti-SARS-CoV-2 assay correlation plots for the Elecsys Anti-SARS-CoV-2 N ECLIA presumptive**-**positive samples tested by the Elecsys S ECLIA and VITROS S ChLIA.** Correlation plots for the Elecsys SARS-CoV-2 S ECLIA and the VITROS Anti-SARS-CoV-2 Total S ChLIA for the: (A) 91 presumptive-positive samples with available volume (62/91 tested; p<0.0001, R^2^=0.9411). (B) 55 confirmed-positive samples with available volume (40/55 tested; p<0.0001, R^2^=0.9359). (C) 28 neutralized-positive samples with available volume (20/28 tested; p<0.0001, R^2^=0.9177). Collection dates for four samples with the highest reactivity observed by a combination of the anti-S, anti-RBD assays and/or with the greatest neutralization activity from Figure 2 are: March 23, April 18 and 23, and May 1, 2020; in all panels these samples are highlighted by month of collection (3 of 4 were available for additional VITROS S testing): March (red), April (green) and May (blue). ECLIA: electrochemiluminescence immunoassay; ChLIA: chemiluminescent immunoassay; S: spike; S/CO: signal-to-cutoff ratio; U/mL: units per milliliter.

**Supplemental Figure 3. Anti-SARS-CoV-2 assay correlation plots for the Elecsys Anti-SARS-CoV-2 N ECLIA presumptive-positive samples tested by the VITROS N ChLIA.** Correlation plots for the Elecsys Anti-SARS-CoV-2 N ECLIA and the VITROS Anti-SARS-CoV-2 Total N ChLIA for the: (A) 91 presumptive-positive samples with available volume (80/91 tested; p<0.0001, R^2^=0.6798). (B) 55 confirmed-positive samples with available volume (50/55 tested; p<0.0001, R^2^=0.6274). (C) 28 neutralized-positive samples with available volume (27/28 tested; p<0.0001, R^2^=0.4542). Collection dates for four samples with the highest reactivity observed by a combination of the anti-S, anti-RBD assays and/or with the greatest neutralization activity from Figure 2 are: March 23, April 18 and 23, and May 1, 2020; in all panels these samples are highlighted by month of collection: March (red), April (green) and May (blue). ECLIA: electrochemiluminescence immunoassay; ChLIA: chemiluminescent immunoassay; N: nucleocapsid; S/CO: signal-to-cutoff ratio; COI: cutoff index.
