# Supplementary figures and images for: Serologic Testing of US Blood Donations to Identify Severe Acute Respiratory Syndrome Coronavirus 2 and Other Coronaviruses, December 2019 to July 2020
Source: Open Forum Infect Dis. 2024 Jun 28;11(7):ofae351. doi: 10.1093/ofid/ofae351 (PMC11257073; doi:10.1093/ofid/ofae351)

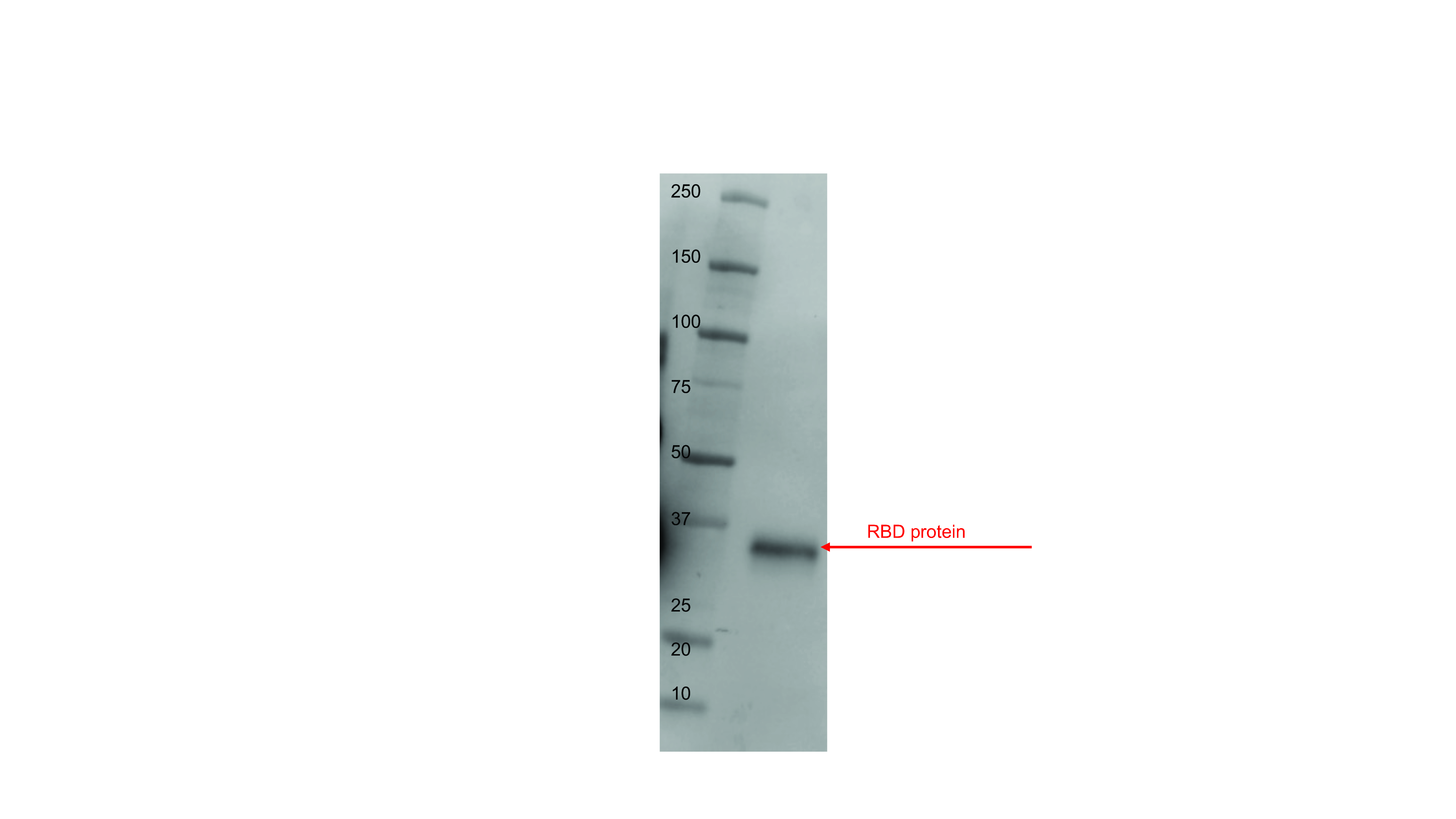

Supplement: ofae351_Supplementary_Data [file ofae351_supplementary_data.zip › SUP FIG 1.tif]

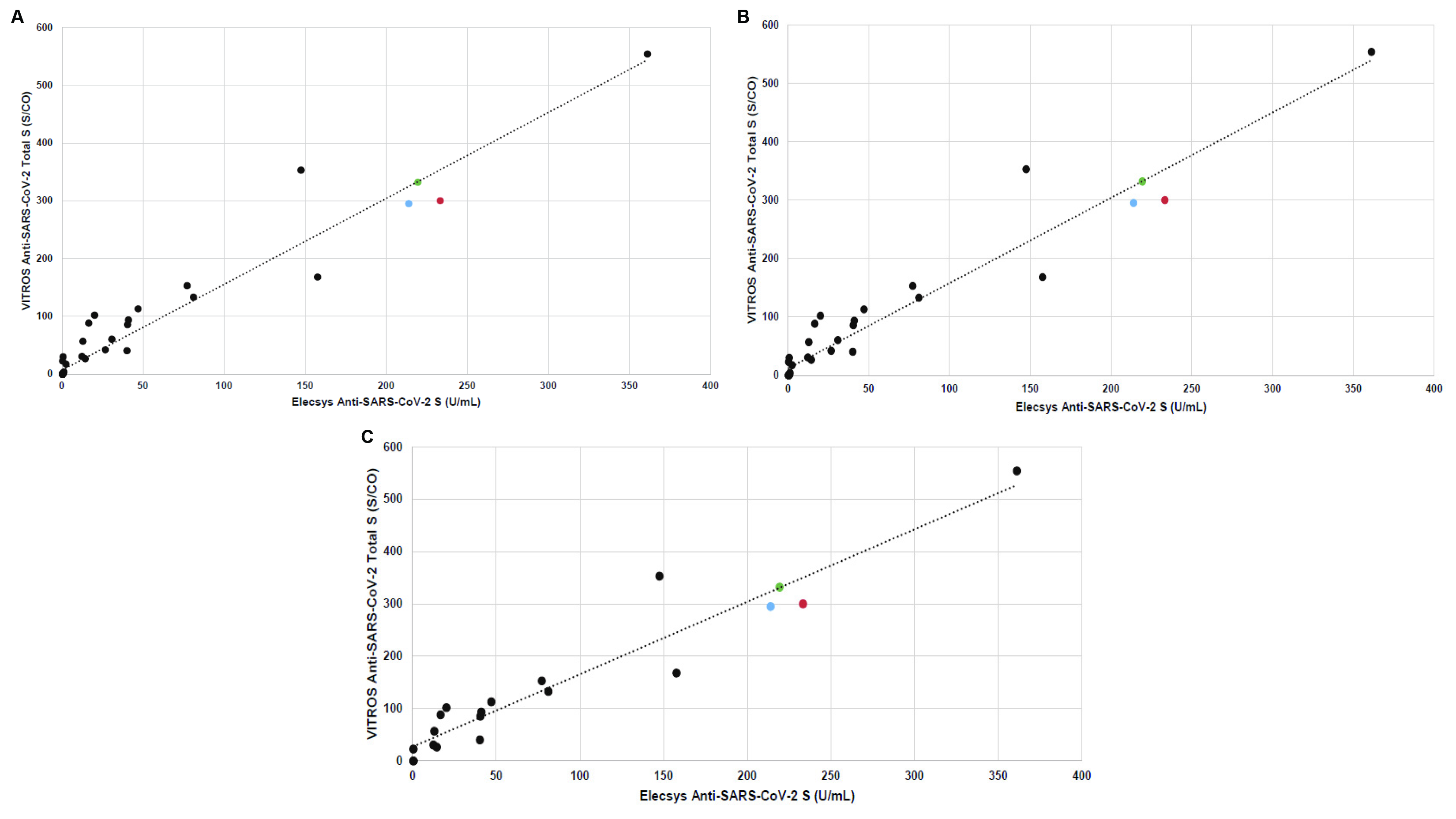

Supplement: ofae351_Supplementary_Data [file ofae351_supplementary_data.zip › Supp Fig 2.tif]

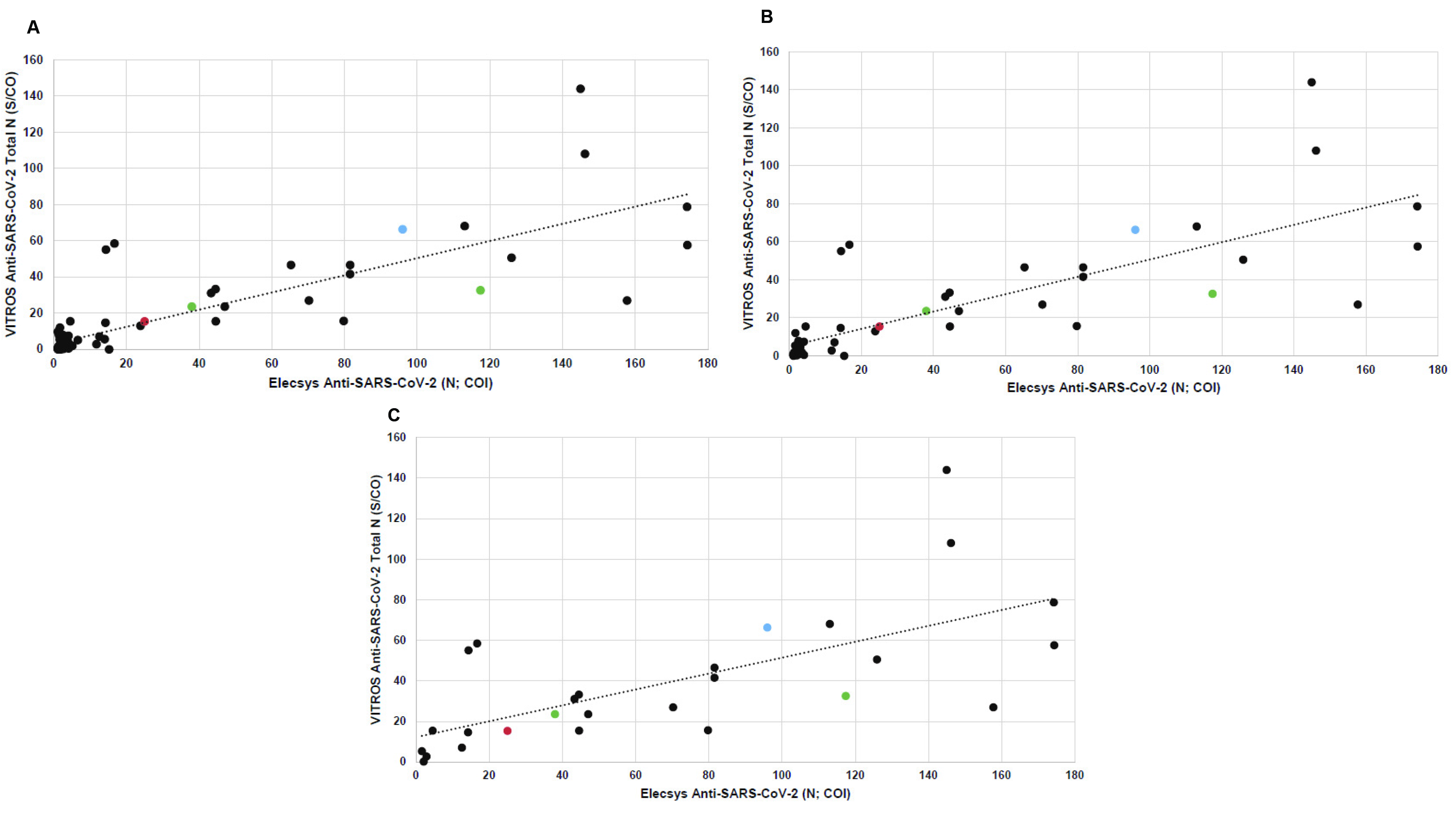

Supplement: ofae351_Supplementary_Data [file ofae351_supplementary_data.zip › Supp Fig 3.tif]
